# Death Following Methyl Alcohol Intoxication: Gross Autopsy and Histological Findings

**DOI:** 10.7759/cureus.65498

**Published:** 2024-07-27

**Authors:** Biliana Mileva, Tihomir Dikov, Metodi Goshev, Mihaela Georgieva, Ivan I Tsranchev, Martin Angelov, Alexandar Alexandrov, Navneet Ateriya, Vesela Ivanova

**Affiliations:** 1 Department of Forensic Medicine and Deontology, Medical University Sofia, Sofia, BGR; 2 Department of General and Clinical Pathology, University Hospital “Alexandrovska”, Medical University of Sofia, Sofia, BGR; 3 Department of Forensic medicine and Deontology, Medical University of Plovdiv, Plovdiv, BGR; 4 Forensic Medicine and Toxicology, All India Institute of Medical Sciences, Gorakhpur, Gorakhpur, IND

**Keywords:** methyl alcohol poisoning, suicide, formic acid, intoxication, toxicology, forensic pathology, methanol poisoning, methanol toxicity, methanol

## Abstract

Methanol, or wood alcohol, is a clear liquid with a weak odor, slightly sweeter than ethanol, which is easily accessible. The last makes it a product of choice for intentional self-harm, severe intoxication, or even suicide. Accidental ingestion and homicidal usage are not exclusions. We present and discuss the case of a man in his 20s who was in continuous alcoholic intoxication until he finally abused with methanol and was admitted to a hospital, where he died six days later. When it comes to intoxication, there are often no apparent findings that could help in determining the cause and manner of death. The last is especially important in cases of delayed death when the toxicology results are negative.

## Introduction

Methyl alcohol poisoning is a serious medical, social, and economic problem, as the consumption of it may cause severe worsening of a person’s health and can lead to death. Most commonly, methanol consumption happens accidentally or intentionally, primarily as a method of suicide. Still, in the literature, some authors have reported homicidal cases due to acute or chronic methanol intoxication [1-3]. Methanol, or wood alcohol, is a clear, colorless, volatile liquid with a weak odor, slightly sweeter than ethanol. It is commonly used in industrial products such as paints, solvents, cleansers, perfumes, antifreeze, automotive windshield washer fluids, and commercial formaldehyde, as well as in the production of illegal alcohol [4,5]. Most fatal cases result from ingestion, less often from inhalation, and exceptionally rarely due to dermal absorption [6-9]. Methanol by itself is a substance with low toxicity. The severe intoxication is due to the action of the products of its metabolism: formaldehyde and formic acid. They are responsible for deteriorating the individual’s health through metabolic acidosis, ocular damage, and central nervous system (CNS) damage [10-12].

The current study was presented as a poster presentation in June 2019 at the XXIX International Scientific Meeting of the Union of Scientists - Stara Zagora, Bulgaria.

## Case presentation

A man in his 20s was admitted to the hospital in a worsening general condition with Glasgow Coma Scale (GCS) 3: non-reactive pupils, complete atony and areflexia, perioral cyanosis, and bradypnea. There was additional data collected from the patient’s parents that he was in severe alcoholic abuse for about two weeks prior to his hospital admission. They explain his behavior of continuous drinking as a reaction to some personal problems. The mother of the deceased man reported that she had found missing from their home a certain amount of methyl alcohol from a bottle with wild chestnuts. The toxicological analysis from the hospital showed high blood methanol levels (4.8‰) and elevated levels of formic acid (25 mmol/l). Immediately after his admission to the hospital, the patient went into clinical death. He was intubated and plugged into life-support systems. Still, despite all the efforts, treatment, and medical care-gastric lavage, hemodialysis, alkalizing, and administration of antidotes, the patient died on the sixth day due to respiratory and circulatory failure. The autopsy was performed two days later.

**Figure 1:**
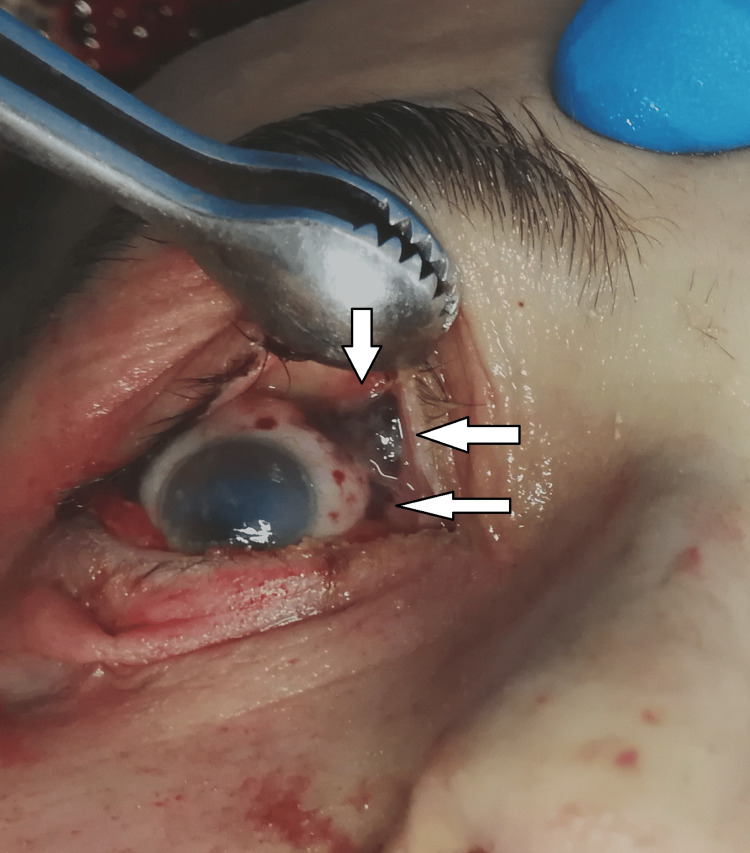
Dot-like dark reddish hemorrhages under the conjunctivae of the right eye

**Figure 2:**
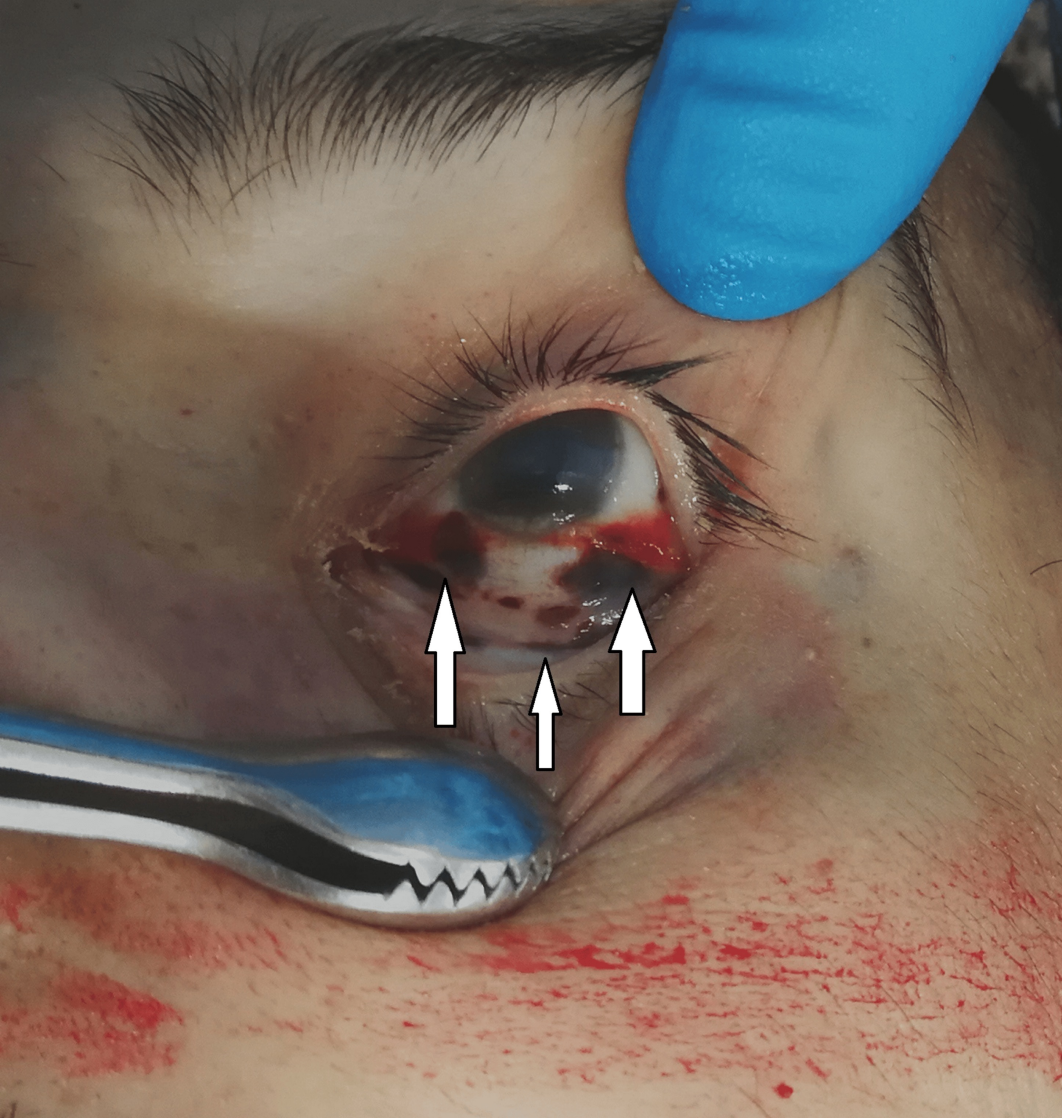
Dot-like dark reddish hemorrhages under the conjunctivae of the left eye

The autopsy started with a thorough external examination of the corpse. The postmortem changes were noted: the lividity was well pronounced, intensive, with purplish color in the dependent not pressed parts of the body - fixed. The muscle stiffening was still present, and the corpse was cold. No traumatic injuries were found. Under the conjunctivae of both eyes, there were limited and merging in places, dot-like, dark reddish hemorrhages. (Figures 1-2).

Massive congestion of the internal organs was noted during the internal examination of the body. The brain was edematous (with widened gyri, narrowed sulci, and flattened surface) and congested, with a generally preserved shape and soft consistency. No hemorrhages or hematomas were noted.

**Figure 3:**
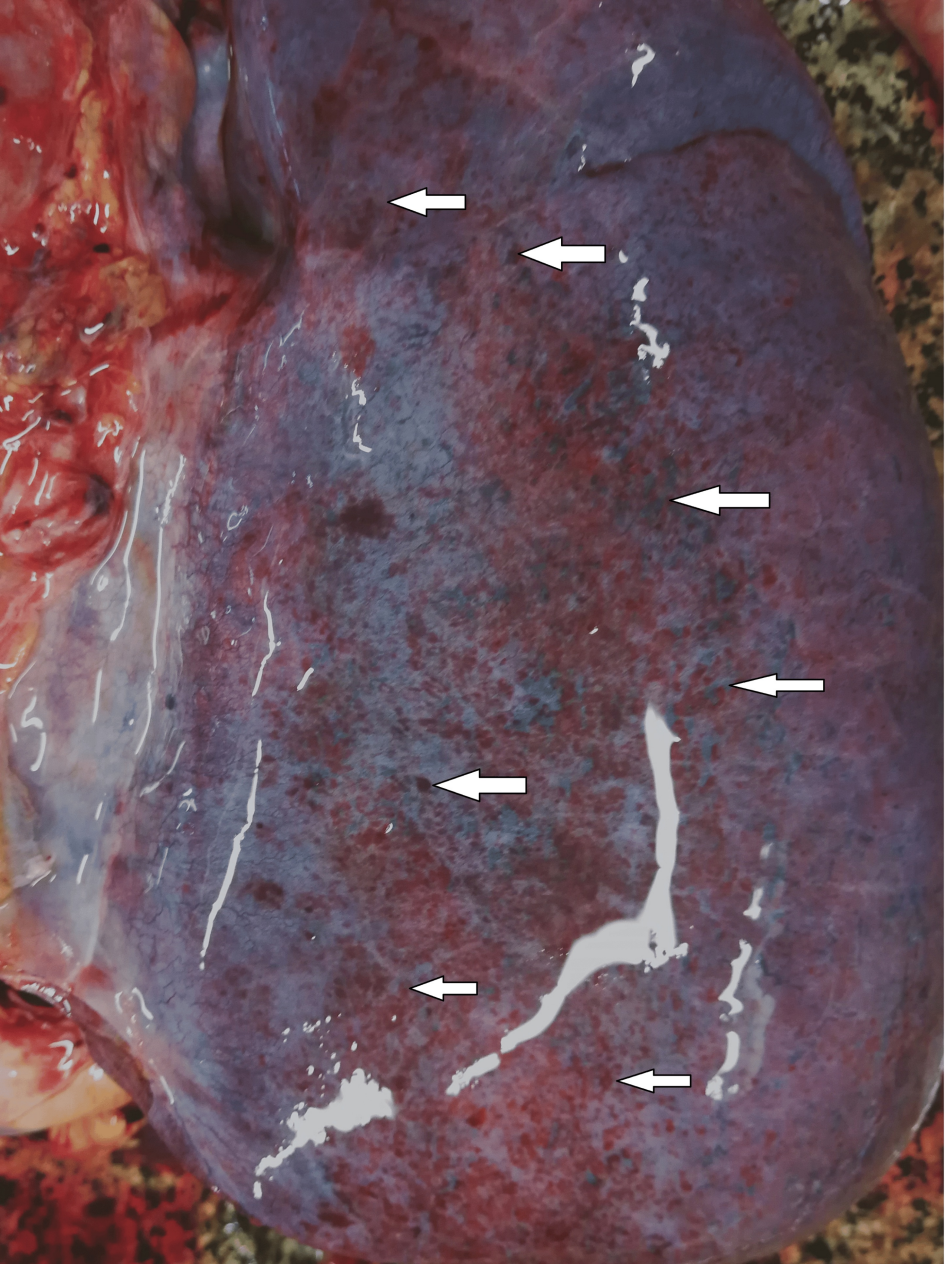
Sub-pleural dot-like hemorrhages

The mucus membrane of the trachea and the bronchi was reddened and covered with scanty, purulent exudation. The lungs were congested and edematous, with massive sub-pleural, merged, dot-like, dark reddish hemorrhages with diameters between 2 and 3 mm (Figure 3).

**Figure 4:**
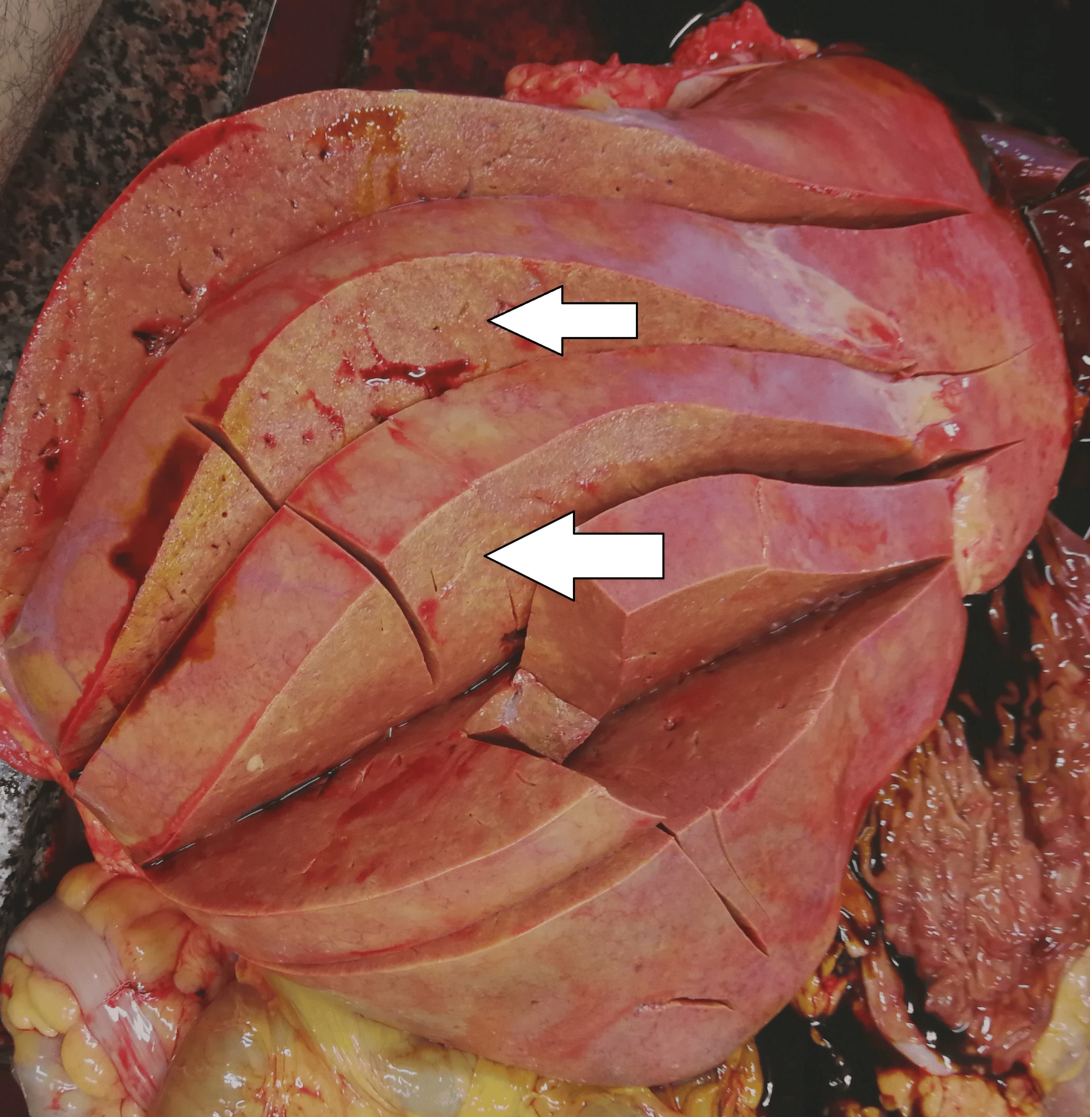
Liver - macroscopic appearance

The heart was enlarged, with mild hypertrophy of the left ventricle. The liver was enlarged, with a smooth capsule and a yellowish color. The cutting surface was the same yellowish color (Figure 4).

**Figure 5:**
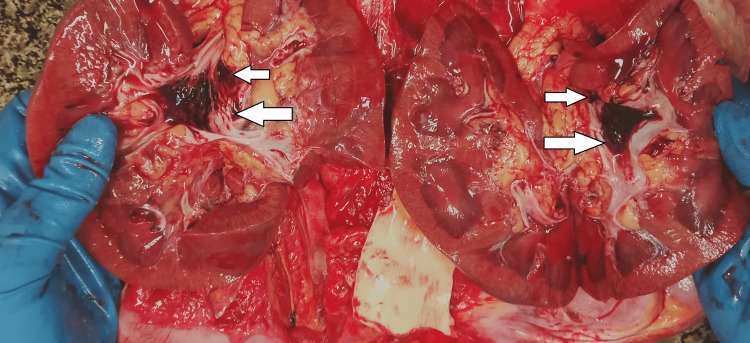
Kidneys - macroscopic appearance - merging hemorrhages under the mucous membrane of the renal pelvis

The kidneys had a smooth surface, with massive merging hemorrhages under the mucous membrane of the renal pelvis (Figure 5).

The spleen had a soft consistency, its capsule was wrinkled, and the cutting surface had autolytic changes. The results from the toxicology analysis were negative for methanol, ethyl alcohol, ethylene glycol, and other drugs.

Tissue samples were taken for further histopathological evaluation and stained with hematoxylin and eosin. The results from the microscopic examination of the examined tissue samples are presented and described in detail in Figures 6-15.

**Figure 6:**
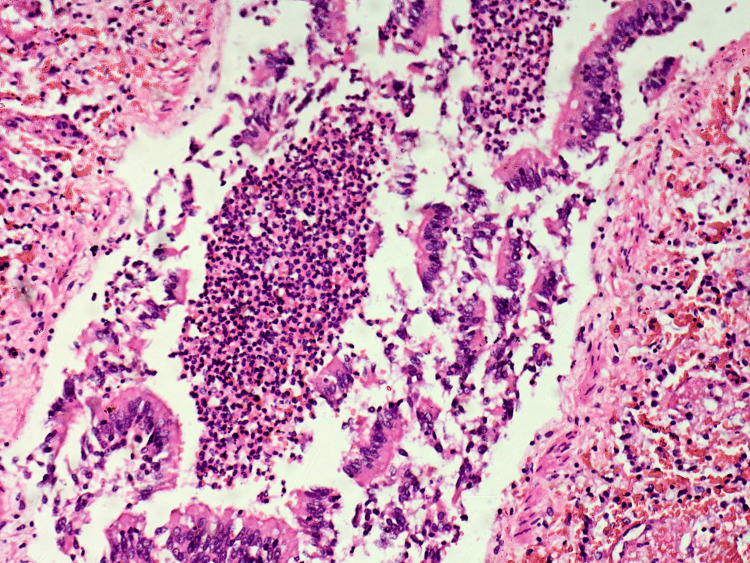
Lung - acute purulent, desquamative bronchitis and bronchiolitis

**Figure 7:**
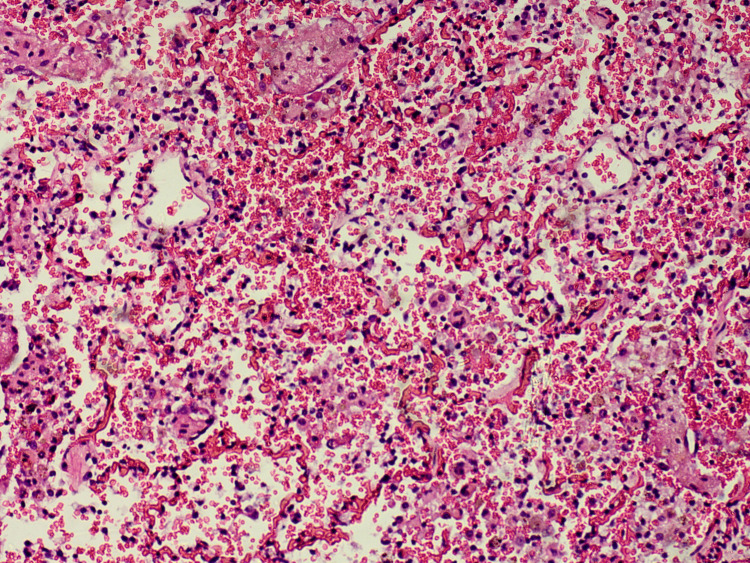
Lung - intense alveolar capillary congestion, intraalveolar hemorrhages

**Figure 8:**
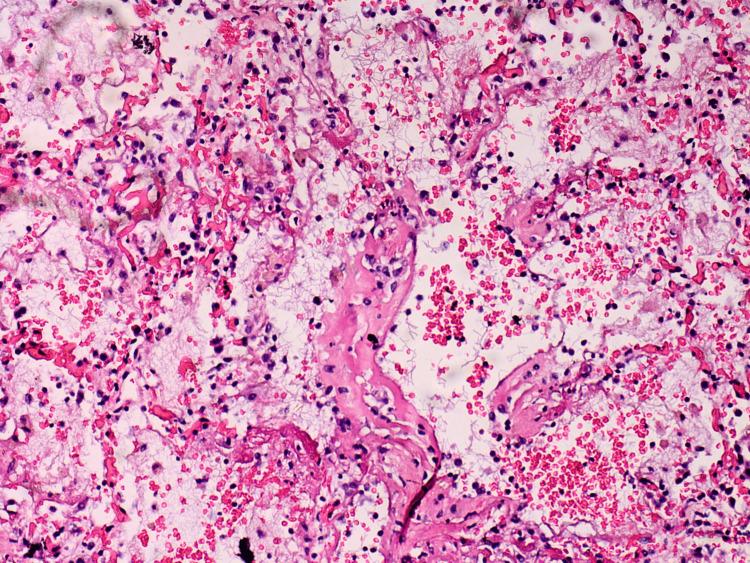
Lung - exudative phase with hyaline membranes

**Figure 9:**
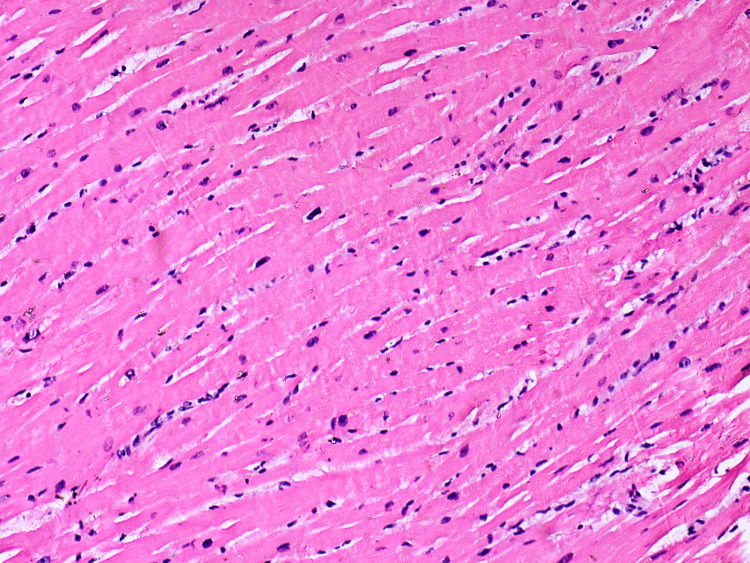
Myocardium - irregular myocardiocyte hypertrophy, coagulative type myocytolisis

**Figure 10:**
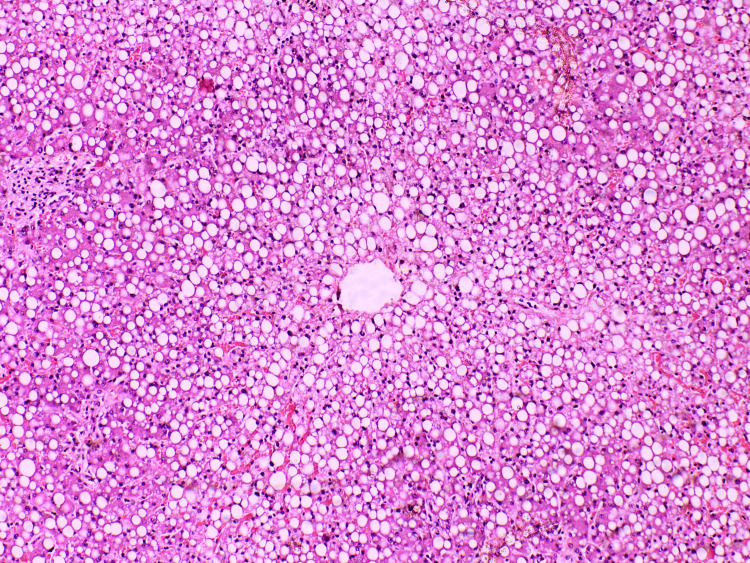
Liver - diffuse, panlobular, isometric, medium-sized fatty degeneration (steatosis)

**Figure 11:**
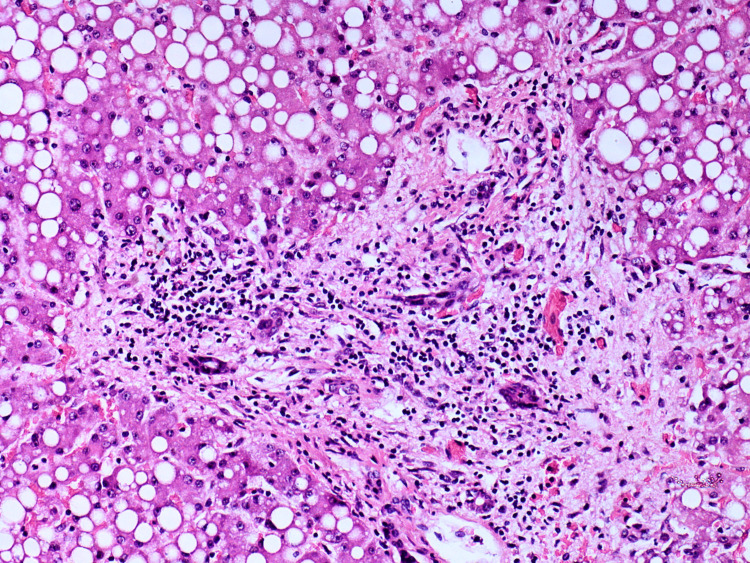
Liver - expended portal tracts due to moderate to heavy inflammatory infiltrate with focuses on lobular aggression, bile duct proliferation

**Figure 12:**
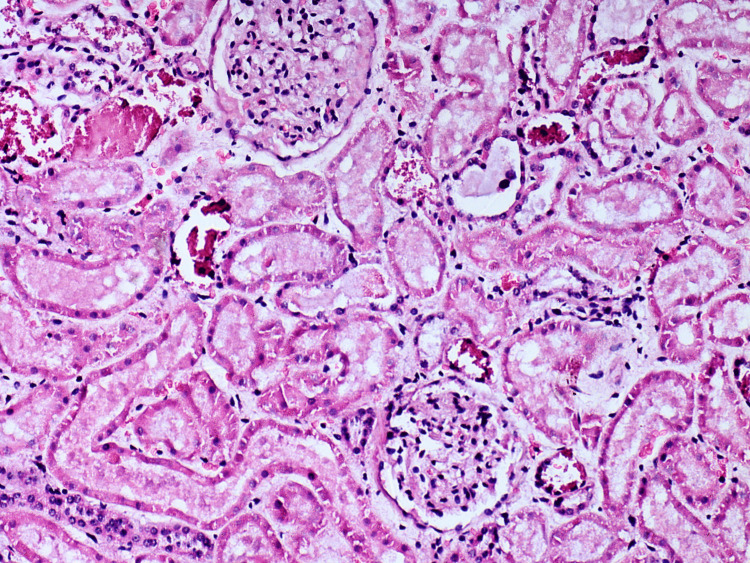
Kidney - acute tubular injury (cellular edema) with intact basal cell membranes, venous congestion

**Figure 13:**
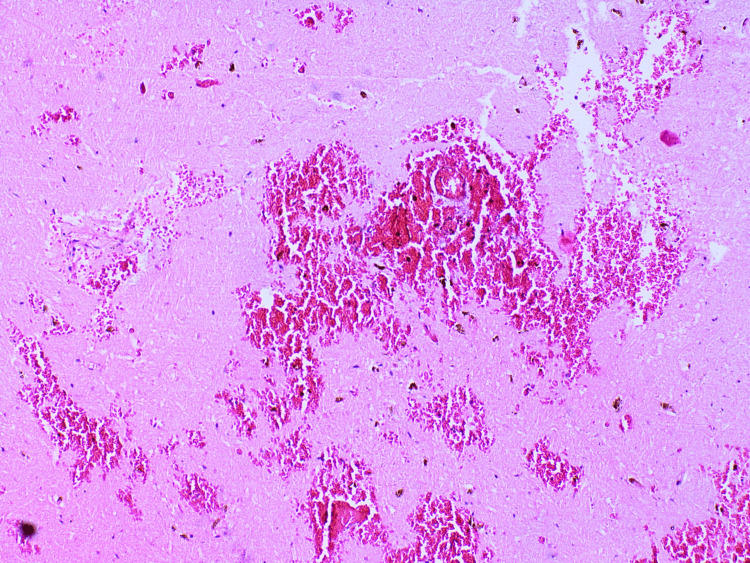
Brain - intense congestion and multiple focuses of fresh hemorrhages

**Figure 14:**
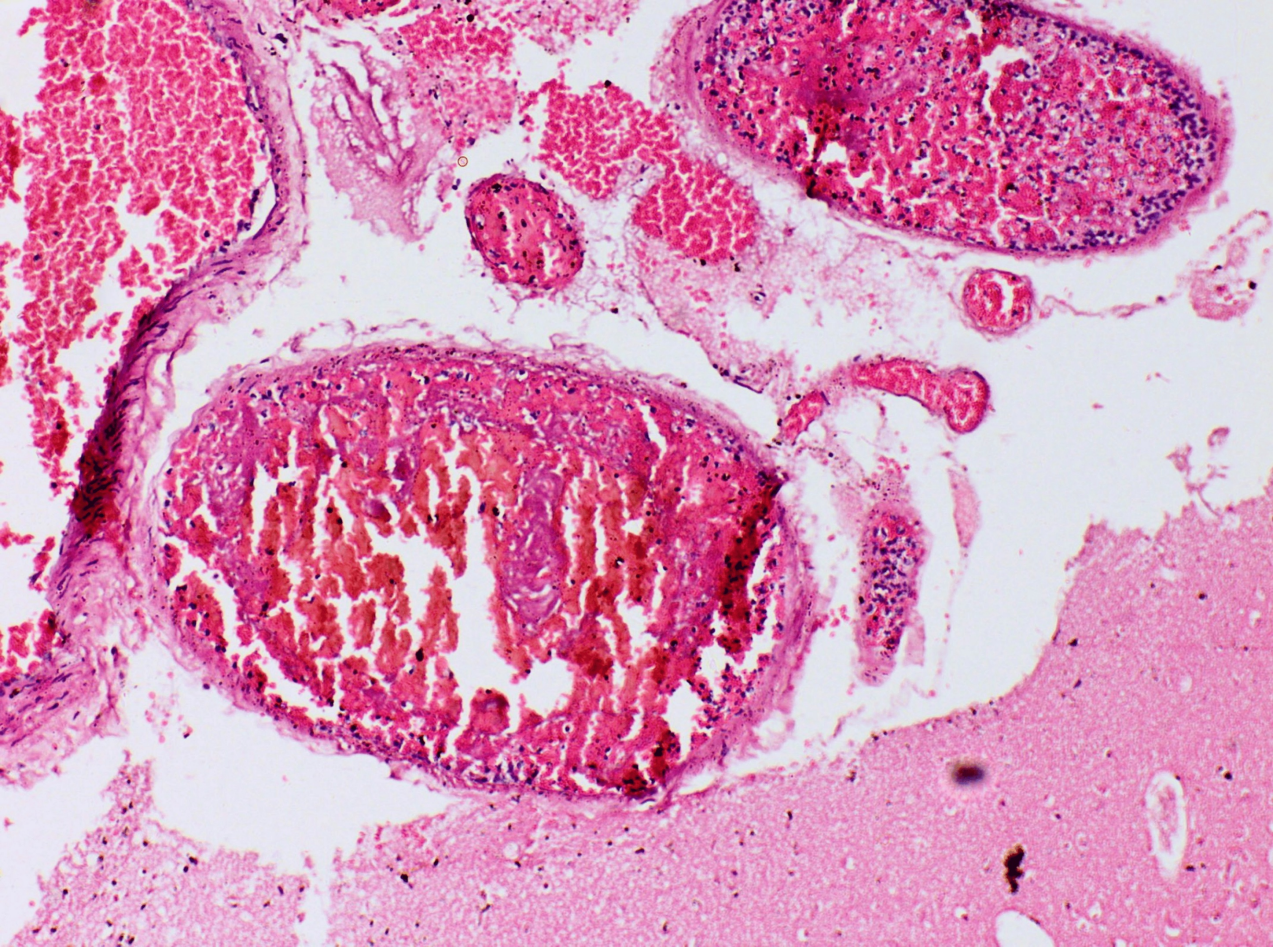
Brain - recently mixed thrombi in small to medium-sized blood venous vessels

**Figure 15:**
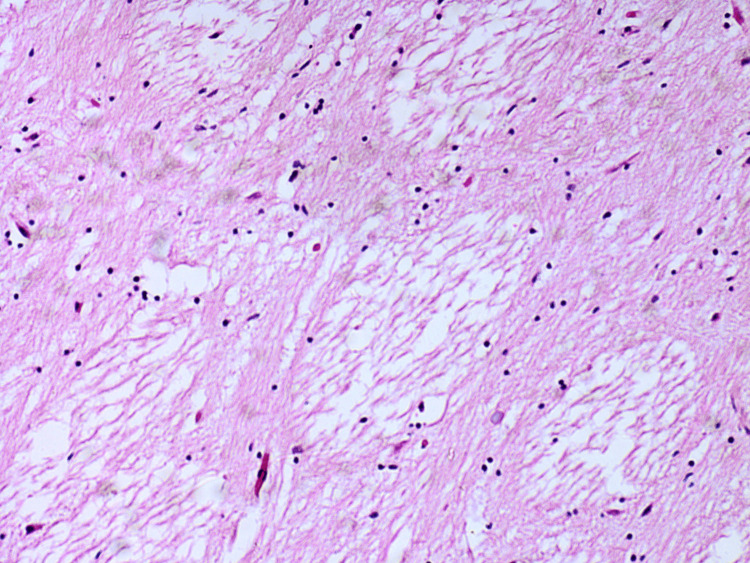
Brain - loosely textured brain tissue due to edema and bordering true coagulative necrosis

The cause of death in the given case was opined to be severe intoxication with methanol and its metabolites (formic acid), leading to severe acidosis, cardiac failure, and anoxic brain injury.

## Discussion

Methanol intoxication has been widely studied and well-known since methanol was recognized as a serious neurotoxin in humans [13]. The most common symptoms observed following methanol ingestion are nausea, abdominal pain, visual disturbances, and mental status changes [13-15]. The onset of symptoms may be visible following a latent period of about 12 to 24 hours after ingestion. In more complicated cases with severe intoxication, methanol consumption may cause delirium, convulsions, coma, cardiopulmonary failure, and death [13-15]. In some sporadic cases, death might be delayed, as reported by Rahimi et al., who describe a case of a 28-year-old man who died 20 days following methanol consumption [16].

Methanol intoxication manifests with some typical macroscopic and microscopic findings. The most common pathological changes observed in the different organs are as follows: brain engorgement of the circulatory system in both the dura mater and the pia-arachnoid-well-pronounced swelling of the brain substance [17]. Bilateral hemorrhagic putaminal necrosis and optic nerve lesions are considered to be two of the most characteristic findings in cases of poisoning with the studied substance. Subcortical, deep white matter lesions, cerebral and cerebellar cortical lesions, and midbrain lesions are observed, too [16,18-20]. Histopathological evaluation of the brain tissue most often shows cerebral congestion and edema. Histopathological assessment of the optic nerve shows edematous changes [21]. Some authors have reported significant demyelination with intra-axonal swelling and organelle destruction [22]. The second target organ that is highly affected by the intoxication, more precisely due to the accumulation of formic acid, is the liver. Commonly observed histopathological findings are micro-vesicular steatosis, macro-vesicular steatosis, hydropic degeneration, feathery degeneration, and hyperemia [5,23]. The kidney damage consists of hyperemia, parenchymal degeneration, tubular injury, tubular necrosis, and hydropic change [17,23]. The authors have reported some changes in the heart: myocardial acute ischemic changes, hyperemia, and hypertrophy. The findings, connected with the lungs, are mainly edema, congestion, and intraalveolar and sub-pleural hemorrhages [23]. Cascallana et al. reported in their study a case of methanol intoxication with severe necrosis of the esophageal and gastric mucosa [24].

Methanol, as a cell toxin, leads to various changes in all organs and systems in the human body [10-13]. The findings associated with the current case report match the ones shown in the literature, although not all were observed macroscopically. For example, the brain showed no apparent findings apart from engorgement and congestion; microscopically, fresh hemorrhages were present. A general pathologic response to the toxic substances as disseminated coagulopathy could be observed under a microscope. This could explain the presence of mixed thrombi in small to medium-sized blood venous vessels observed in the microscopic examination of the brain tissue. The prolonged hospital stay can explain the established lung inflammatory changes. On the other hand, the acute lung injury described by the exudative phase with hyaline membranes could be a sign of shock or could be associated with diffuse alveolar damage. The microscopic kidney changes might be linked to toxic acute tubular necrosis without tubular rhexis. The changes observed in the liver might be connected to both methanol consumption and prolonged alcohol consumption. Additionally, some features corresponded to severe tissue hypoxia as a result of severe metabolic acidosis, which the action of the methanol metabolite (formic acid) could explain: multiple dot-like hemorrhages under the serous mucosa of the internal organs and the conjunctiva of the eyes. The cause of death was attributed to severe brain swelling with paralysis of the centers of the respiratory and cardiovascular systems as a complication of acute intoxication with methyl alcohol and its metabolites.

Based on the findings described in the literature and the ones observed in the presented case, we suggest a classification of the macroscopic and microscopic findings consisting of two main groups as follows: characteristic findings for methanol intoxication and supportive findings or borderline findings for intoxication with methanol and its metabolites (formic acid). In the first group, we include only the findings that are typically and mainly associated with such type of intoxication - the pathological changes observed in the brain and optic nerve - bilateral hemorrhagic putaminal necrosis and optic nerve lesions, subcortical white matter lesions, cerebral, cerebellar cortical, and midbrain lesions [16-20]. In the second group, we include again findings that are typically observed and associated with cases of methanol intoxications but might be linked to other pathological conditions as well - cerebral congestion and edema, presence of thrombi in the small or medium-sized blood venous vessels, micro- and macro-vesicular steatosis of the liver, hydropic degeneration, feathery degeneration and hyperemia of the liver, toxic acute tubular injury, parenchymal degeneration, hyperemia, and hydropic change of the kidneys [5,17,23]. Here, we might also include the multiple dot-like hemorrhages under the serous mucosa of the internal organs and the conjunctiva of both eyes [23].

To make a precise and accurate diagnosis, especially in cases of intoxication, the forensic pathologist needs to be provided with all the information concerning the case - information from the place where the death occurred, testimonies of the witnesses, medical documentation from the hospital stay when there is a survived period, toxicology report, and histopathological evaluation of the tissues samples taken during the autopsy of the deceased.

## Conclusions

The tragic consequences of methyl alcohol intoxication underscore the critical importance of public awareness, education, and stringent regulatory measures. The case of death following methyl alcohol ingestion serves as a poignant reminder of the severe health risks associated with the consumption of adulterated or improperly labeled substances. Although such cases are often seen in forensic practice, this case shows the importance of detailed and thorough analysis since not all “typical findings” might be seen macroscopically. Furthermore, healthcare professionals should remain vigilant in recognizing the signs and symptoms of methyl alcohol poisoning, ensuring timely diagnosis and intervention. Improved medical education and accessibility to antidotes can play a crucial role in reducing the mortality rates associated with such intoxication.
